# Fatal Human Infection with Rabies-related Duvenhage Virus, South Africa

**DOI:** 10.3201/eid1212.060764

**Published:** 2006-12

**Authors:** Janusz T. Paweska, Lucille H. Blumberg, Charl Liebenberg, Richard H. Hewlett, Antoinette A. Grobbelaar, Patricia A. Leman, Janice E. Croft, Louis H. Nel, Louise Nutt, Robert Swanepoel

**Affiliations:** * National Institute for Communicable Diseases, Sandringham, South Africa;; † Durbanville Mediclinic, Cape Town, South Africa;; ‡ University of Stellenbosch, Tygerberg, South Africa;; § University of Pretoria, Pretoria, South Africa

**Keywords:** Duvenhage virus, rabies-related lyssavirus, fatal human infection, South Africa, dispatch

## Abstract

Duvenhage virus was isolated from a patient who died of a rabieslike disease after being scratched by a bat early in 2006. This occurred ≈80 km from the site where the only other known human infection with the virus had occurred 36 years earlier.

The genus Lyssavirus within the family Rhabdoviridae currently includes rabies virus (RABV) (genotype 1) and 6 rabies-related viruses: 3 from Africa, Lagos bat virus (LBV) (genotype 2), Mokola virus (MOKV) (genotype 3), and Duvenhage virus (DUVV) (genotype 4); European bat lyssaviruses 1 and 2 (EBLV1 and 2) (genotypes 5 and 6); and Australian bat lyssavirus (ABLV) (genotype 7) (*1*). Strains of RABV (genotype 1) undergo genetic adaptation to particular animal hosts so that within specific areas the disease is manifested and transmitted predominantly by 1 host species. The canid, or dog, biotype of RABV is the most widely distributed in the world. In South Africa, RABV is transmitted by dogs and jackals in the northern region of the country, by dogs in the eastern region where most cases of human rabies occur, and by bat-eared foxes in the western region. In addition, an indigenous herpestid biotype of RABV (genotype 1) is transmitted by mongooses (Herpestidae) on the interior plateau of South Africa. This biotype does not spread readily to dogs but causes occasional cases of rabies in dogs, cats, humans, and more frequently, cattle and sheep (*2*).

RABV (genotype 1) has never been isolated from bats outside North and South America, but rabies-related viruses have been isolated from bats elsewhere. In Africa, LBV and DUVV are associated with bats, but MOKV is uniquely associated with shrews and rodents, not bats. Fifteen isolations of LBV have been reported, including 8 from fruit bats and a cat in KwaZulu-Natal Province of South Africa, but the virus has never been associated with human disease (*2**,**3*). MOKV has been isolated from shrews, rodents, cats, and a dog in Africa and from 7 cats with rabies-like disease in KwaZulu-Natal and Eastern Cape provinces of South Africa (*2*). The virus is believed to have caused rabieslike disease in 2 persons in Nigeria in 1969 and 1971, shortly after its initial discovery in shrews in 1968, but no cases of human infection have subsequently been recognized (*4**,**5*). DUVV was discovered in 1970 when it caused fatal rabieslike disease in a person bitten by an unidentified insectivorous bat ≈150 km northwest of Johannesburg, South Africa (*6*). In 1981, the virus was isolated from what is believed to have been a Miniopterus schreibersi insectivorous bat caught in daylight by a cat in Makhado town (formerly Louis Trichardt) in Limpopo Province, South Africa, and in 1986 the virus was recovered from an insectivorous bat, Nycteris thebaica, trapped in a survey in Zimbabwe (*7**,**8*).

## The Study

DUVV infection was recently confirmed in a 77-year-old man with type 2 diabetes who was scratched on the face by what appears to have been an insectivorous bat in February 2006 in North West Province, South Africa, ≈80 km from the location where the first DUVV infection occurred 36 years earlier. The bat flew into a room at night, landed on the man's spectacles while he was attempting to chase it out, and scratched his face as he brushed it off. The bat did not appear to have bitten him, and it escaped after the incident. He did not seek medical care, and thus no postexposure treatment was given. He became ill at home in Cape Town 27 days later and received treatment for influenzalike illness. He slept most of the following day, had hallucinations that night, and was admitted to a hospital on the third day of illness. On admission, he had a fever (40°C), tachycardia, neck and general limb rigidity, hyperreflexia, facial fasciculation, and involuntary grimacing. Within 24 hours generalized tonic-clonic seizures had developed with status epilepticus supervening. These necessitated intubation, sedation, and mechanical ventilation. He died on day 14 of his illness.

Heminested reverse transcription–PCR was performed as previously described (*9*) with modified forward primer JW12 (*10*). This procedure detected lyssavirus nucleic acid in saliva taken on day 10 of illness and in brain tissue collected postmortem. Nucleotide sequencing of the PCR products and phylogenetic analysis performed as previously described (*11*) confirmed the identity of the agent as DUVV (Figure), and live virus was isolated from saliva and brain tissue by mouse inoculation. Immunofluorescence tests with antirabies conjugate prepared to be cross-reactive with the African rabies–related viruses (Onderstepoort Veterinary Institute, Pretoria, South Africa) showed small and sparse inclusion bodies in impression smears of the cortex, hippocampus, thalamus, medulla, and cerebellum. Histopathologic examination of sections from the cortex, hippocampus, thalamus, hypothalamus, midbrain, pons, medulla, and cerebellum showed polioencephalitis affecting predominantly the diencephalon and brainstem and involving varying degrees of neuronopathy, neuronal loss, astrocytosis, parenchymal and perivascular lymphocytic infiltration with CD45 immunopositivity, sparse macrophage activation, and axonal spheroid formation. No nuclear or cytoplasmic inclusions were observed.

**Figure:**
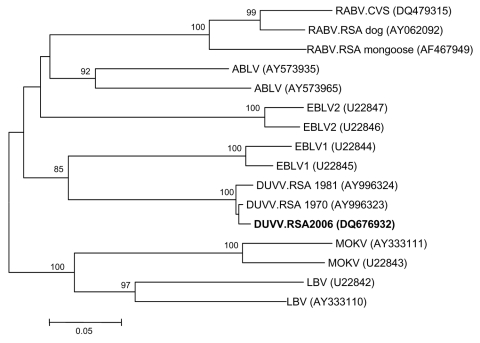
Neighbor-joining tree relating a 372-bp nucleotide sequence of the nucleoprotein gene of the recent Duvenhage virus (DUVV) isolate (boldface) to representative sequences of the known lyssavirus genotypes, including South African dog and mongoose isolates and the reference challenge virus strain (CVS) of rabies virus (RABV) (GenBank accession nos. are indicated in parentheses). Bootstrap values were determined by 1,000 replicates. ABLV, Australian bat lyssavirus; EBLV, European bat lyssavirus; MOKV, Mokola virus; LBV, Lagos bat virus.

## Conclusions

The ability to distinguish between various lyssaviruses and monitor their relative distribution and prevalence has important implications for implementation of control measures. It was recognized in 1932 that mongoose-associated rabies in South Africa differs from classic dog rabies (*2*). Although an inadequately characterized lyssavirus was isolated from a bat trapped in a survey in 1963, before the existence of rabies-related viruses was known, awareness of lyssaviruses other than rabies viruses dates from the identification of DUVV in 1970 and was followed by detection of LBV and MOKV in South Africa (*6**,**7**,**12*). Routine differentiation of diagnostic isolates became feasible with the availability of monoclonal antibodies during the 1980s and the subsequent introduction of molecular epidemiology techniques (*7**,**13**–**15*). Although instances of persons seeking rabies prophylaxis after exposure to bats have been reported, the recent case of DUVV infection constitutes only the second known instance of a person in South Africa with lyssavirus infection after such an encounter. Nevertheless, it is clear that rabies-related viruses are widely endemic in South Africa and that active investigation of the bat-associated lyssaviruses is warranted.
